# Parity and Risk of Stroke among Chinese Women: Cross-sectional Evidence from the Dongfeng-Tongji Cohort Study

**DOI:** 10.1038/srep16992

**Published:** 2015-11-26

**Authors:** Yanmei Zhang, Lijun Shen, Jing Wu, Guiqiang Xu, Lulu Song, Siyi Yang, Yaohua Tian, Jing Yuan, Yuan Liang, Youjie Wang

**Affiliations:** 1 MOE Key Lab of Environment and Health, School of Public Health, Tongji Medical College, Huazhong University of Science & Technology, Wuhan, China; 2 Department of Maternal and Child Health, School of Public Health, Tongji Medical College, Huazhong University of Science & Technology, Wuhan, China; 3 Department of Social Medicine, School of Public Health, Tongji Medical College, Huazhong University of Science & Technology, Wuhan, China

## Abstract

Epidemiological studies have investigated the association between parity and the risk of stroke, but the results have been inconsistent. The objective of this study was to assess the association among middle-aged and older Chinese women. We used data from the Dongfeng-Tongji Cohort Study. In total, 14,277 women were included in the analysis. Participants were classified into four groups according to parity. Stroke cases were self-reported during face-to-face interviews. Multivariable logistic regression models were used to investigate the association between parity and the risk of stroke while controlling for potential confounders. The prevalence of stroke among the study subjects was 2.7% (380 of 14,277). In the fully adjusted model, women who had experienced two, three, or four or more live births had 1.24 times (95% CI, 0.85–1.81), 1.97 times (95% CI, 1.30–2.98) and 1.86 times (95% CI, 1.14–3.03), higher risk of stroke, respectively, compared with women who had experienced one live birth. High parity was associated with an increasing risk of stroke in the present study. Further longitudinal studies are needed to confirm the association and to explore the unclear mechanism underlying the link between parity and stroke risk.

Stroke is a global health problem; it is the second most common cause of death and a leading cause of adult disability worldwide^1^. In China, stroke is the first cause of death and disability-adjusted life-years lost (DALYs lost) according to the global burden of disease (GBD) 2010 and the WHO’s health statistics and information systems^2^,^3^. Stroke remains one of the most devastating of all neurological conditions. Globally, stroke accounts for approximately 5.9 million deaths annually, with 102.2 million disability-adjusted life-years lost according to the GBD 2010 study^3^. Moreover, the number of patients with stroke will increase in the future because of demographic changes and the inadequate control of major risk factors for stroke. Therefore, it is crucial to identify the risk factors of stroke for the prevention of disease and its complications.

An epidemiological study has shown that stroke has a greater effect on women than on men because women experience more events and are less likely to recover^4^. The mechanisms for these sex-specific differences are unclear, and it has been argued that female reproductive factors may play a role in the development of stroke. Pregnancy induces dramatic alterations in the physiology, metabolism, and lifestyle. All of these changes may have a long-term influence on women’s prospective health^5^.

Parity has been reported as a long-term risk factor for coronary heart disease (CHD)^6^,^7^. Because of shared factors between CHD and stroke, it may be inferred that the risk of stroke also increases as parity increases. A number of studies have reported positive associations^7^,^8^,^9^ between parity and stroke risk. Conversely, several studies have reported a protective effect of parity on stroke risk^10^,^11^.

With regard to the controversy, the association between parity and risk of stroke remains to be clarified. Therefore, in this study we aimed to examine the association between parity and risk of stroke in middle-aged and older Chinese women based on the data from the Dongfeng-Tongji Cohort Study.

## Results

### Baseline characteristics

Table 1 presents the subjects’ baseline characteristics according to parity categorized into four groups. Among the 14,277 eligible participants, 380 (2.7%) reported a physician-diagnosed history of stroke. Age increased steadily across parity categories (*P* for trend < 0.001). The subjects with higher parity tended to have higher BMIs (*P* for trend <0.001). The incidence of stroke was positively related to parity, at 1.2%, 2.2%, 4.4%, and 5.2% for women who had experienced one, two, three, or four or more live births, respectively. Interestingly, family members of women with higher parity were less likely to suffer from stroke (*P* for trend < 0.001). Compared with women who had experienced one live birth, women with higher parity (two, three, or four or more live births) were more likely to report physician-diagnosed hypertension and diabetes. Women with higher parity tended to have a lower passive smoking rate and lower educational level (*P* for trend < 0.001). However, they had a higher smoking rate themselves than did those with a lower parity (*P* for trend < 0.001). In addition, higher parity women had a lower rate of adopting contraceptive methods or hormone replacement therapy (*P* for trend < 0.001). All differences for the listed variables except physical activity were statistically significant among different parity groups (*P* < 0.05).

### Parity and stroke risk

Table 2 presents the results of the unadjusted and multivariate adjusted logistic regression models. For each model, the ORs and 95% CIs of stroke for the parity categories are presented. We used five logistic regression models for the evaluation of the relationship between parity and risk of stroke, to control for the major confounders of stroke. Model 1 (the univariate model) showed that women who had experienced two (OR = 1.88; 95% CI, 1.36–2.59), three (OR = 3.82; 95% CI, 2.79–5.23), or four or more live births (OR = 4.59; 95% CI, 3.29–6.39) had a significantly higher risk of stroke compared with women who had experienced one live birth. The results of Model 2 indicated that higher parity (three live births: OR = 2.28; 95% CI, 1.58–3.31; or four or more live births: OR = 2.28; 95% CI, 1.52–3.44) was associated with an increased risk of stroke, after adjustment for education, marital status, smoking status, alcohol consumption, passive smoking, family history of stroke, menopause status, history of contraceptive use, history of hormone replacement therapy, hypertension, diabetes, physical activity, and menopause age. These covariates have previously been reported to be associated with the risk of stroke^12^,^13^,^14^,^15^,^16^,^17^. As abortion has a biological process similar to live birth, we added it into the above model. In Model 3, we still observed a significantly higher risk of stroke among women with higher parity (three live births: OR = 2.31; 95% CI, 1.59–3.34; or four or more live births: OR = 2.31; 95% CI, 1.53–3.48), compared with those who had experienced one live birth. Model 4 made an additional adjustment for age, which is a major risk factor for stroke^18^, and is closely associated with increasing parity. The ORs attenuated but remained statistically significant, ranging from 1.98 (95% CI, 1.31–2.98) for women who had experienced three live births to 1.87 (95% CI, 1.16–3.04) for women who had experienced four or more live births. Finally, in the fully adjusted model (Model 5), that included BMI, a main known risk factor for stroke^12^,^19^, the ORs were 1.97 (95% CI, 1.30–2.98) for women who had experienced three live births and 1.86 (95% CI, 1.14–3.03) for women who had experienced four or more live births, compared with those with one live birth.

### Parity and stroke prevalence

**Figure 1:**
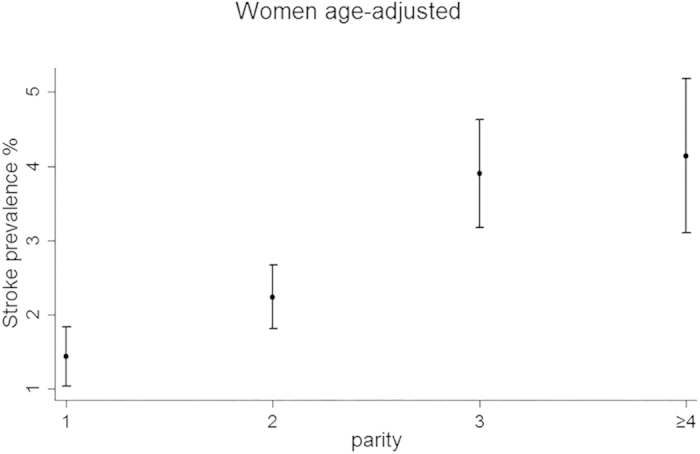
Presents the age-adjusted prevalence of stroke across parity groups, with the circles representing point estimation values and the ranges (including each point) representing 95% confidence intervals.

**Figure 2:**
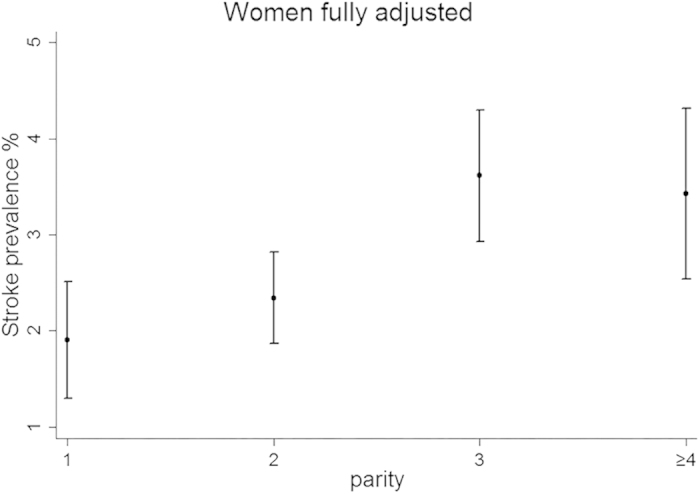
Presents the fully-adjusted prevalence of stroke in women by parity. Every circle represents the point estimation value of stroke prevalence and every range line represents the 95% confidence interval.

The age-adjusted and fully adjusted prevalence of stroke is presented by parity in Figs 1 and 2 respectively. In Fig. 1, the age-adjusted prevalence of stroke was higher for each increasing parity group. The fully adjusted prevalence of stroke (Fig. 2) increased from the one live birth group to the three live births group, but declined slowly in the group with four or more live births. However, the prevalence of stroke was still higher among those with four or more live births than among those with one or two live births.

## Discussion

In this large, population-based study, we found that higher parity was associated with a significantly elevated risk of stroke among middle-aged and older Chinese women. After controlling for potential confounders, the association attenuated but remained statistically significant for women who had experienced three or more live births and those who had experienced one live birth, suggesting that higher parity is a risk factor for stroke in this population of Chinese women.

In the current study, women who had experienced one live birth were taken as the reference group. Nulliparity may reflect an inability to conceive or complete a pregnancy because of health factors such as polycystic ovary disease among Chinese women^20^, a known risk factor for stroke. It was reported that there were significant differences between nulliparous women and parous women of physiology and lifestyle^21^,^22^. Furthermore, in China, the nulliparous women are mainly because of chronic disease or reproductive disease, which may confound the association between nulliparity and risk of stroke^23^. Therefore, nulliparous women were excluded from the data analysis to avoid potential bias in our present study.

The findings of our study are consistent with those of previous studies concerning the association between greater number of pregnancies and greater risk of stroke in women beyond typical childbearing age^8^. For instance, an analysis of women aged 45 to 74 years in the first National Health and Nutrition Examination Survey Epidemiology Follow-up Study reported that women who had six or more pregnancies had a 70% higher risk of stroke compared with those who had never been pregnant^8^. Beral *et al.*, in an earlier British study of middle-aged and elderly women, demonstrated a higher mortality rate from cerebrovascular disease in parous women than in nulliparous women^24^. A recent Chinese study showed that women who had five or more children had an increased risk for all types of incident stroke, with a subgroup comprised of ischemic stroke, intracranial hemorrhage, and undefined stroke^9^. The two studies mentioned above found that only grand parity was associated with a higher risk of stroke. However, in the present study, we found that women who had experienced three or more live births had a higher risk of stroke compared with those who had one live birth, after fully adjusting for potential risk factors. In addition, there are reports of positive associations between parity and both carotid atherosclerosis and carotid intima media thickness, which are strong predictors of stroke^25^,^26^. In contrast to these findings, several studies have shown a decline in the risk for subarachnoid hemorrhage, a subtype of stroke, with increasing parity^10^,^11^,^27^. A study conducted by Mhurchu *et al.*^28^ found no association between parity and risk of subarachnoid hemorrhage. In the present study, we observed that the risk of stroke increased as the number of live births increased.

The biologic mechanism underlying the link between parity and stroke has not been well understood. Pregnancy itself is a risk factor for hemorrhagic stroke^29^,^30^, and the experience of delivery has been reported to increase the risk of intracranial hemorrhage^31^. Multiparity is a known risk factor for pregnancy-induced hypertension^32^, which may increase the risk of stroke including ischemic stroke and hemorrhagic stroke in the later life^33^,^34^. Repeated pregnancies and deliveries may induce physical and psychological stress and also add strain to the cardiovascular system in women with high parity^35^. During pregnancy, hemodynamic changes and oxidative stress may induce decreased vascular resistance, and during delivery, vascular tension may also cause vessel weakening or aneurysms^36^, which may induce hemorrhage stroke. Throughout pregnancy, venous compliance increases, which steers the body towards decreased blood flow and increased venous stasis. The hypercoagulability induced by increased venous stasis could cause cerebral venous thrombosis, which is a significant cause of ischemic stroke. Higher parity is associated with increased carotid atherosclerosis, a well known cause of ischemic stroke^37^. Accumulating evidence suggests that parity is associated with a higher risk of all-cause mortality later in life, and especially with cardiovascular and cerebrovascular mortality^38^. As stroke itself is a kind of cardiovascular disease, the relationship between parity and stroke observed in our study may help to explain the association between parity and cardiovascular disease to some extent.

Pregnancy is a very important period for women, and may result in obesity and postpartum weight retention, which are of crucial importance to a woman’s health in later life^5^,^39^. In China, pregnant women are regarded as the most important people in most families, and they usually have an unhealthy diet of excess nutrition and little engagement in physical activity. Lack of exercise and a high-calorie diet are closely related to increased risks of weight gain and obesity, which were important risk factors for stroke in later life^5^,^12^,^40^. A Chinese study reported that the average pregnancy weight gain was 17.1 ± 4.9 kg among 16,460 Chinese women^41^, a figure, much higher than the recommended amount of weight gain^42^. In the present study, the association between parity and stroke risk was attenuated but remained statistically significant after adjustment for BMI. It was reported that high body-mass index (BMI) was a risk factor for ischemic stroke but not for hemorrhagic stroke^43^. Therefore, BMI may contribute to explaining the association between parity and ischemic stroke risk.

The above-mentioned adverse changes induced by high parity provide biological plausibility for the increased risk of stroke associated with increasing parity in women. While a study conducted by Zhang *et al.*^9^ showed a significant positive association between number of children and prevalence of stroke not only in women, but also in their husbands. This suggests that explanations other than the biological consequences of pregnancy may also need to be considered when interpreting study results on high parity and stroke risk. Childrearing may increase anxiety levels because of increased responsibilities, sleep deprivation, and financial and occupational stress, and it has also been associated with adverse changes in lifestyle such as physical inactivity and unhealthy dietary habits^6^,^44^. These childrearing-related anxiety, stress, and adversely changed lifestyle factors may contribute notably to the elevated risk of stroke associated with high parity. In the current study, we did not obtain corresponding information on men particularly their spouses.

Our study has several strengths. A large sample size, structured questionnaires, standardized medical examinations, laboratory measures and accurate measures of potential confounders were used in the study, and information regarding demographics, reproductive health factors, lifestyle behaviors, history of disease, and medical examination results were available and valid in our study. Additionally, our interviewers were blind to the objectives of the research, and all were well trained before conducting the study. Therefore, to our knowledge, ascertainment bias was unlikely in our study.

This study has several limitations that need to be acknowledged. First, our data were cross-sectional. Although we demonstrated that higher parity was associated with risk of stroke in the present study, causal and temporal associations could not be ideally inferred. However, it is reported that the mean onset age of stroke was 64.9 ± 7.0 years old in Chinese women^45^ and the mean age for childbirth in China was 28.2 years^46^. Therefore, we could infer the vast majority of women had completed the childbearing before the onset of stroke. In our opinion, parity is likely a causal factor for the development of stroke. Second, stroke cases in the present study were obtained from self-reports without further verification by review of medical records, which may result in potential bias. However, several studies had reported that level of agreement between medical records and self-reporting of stroke was pretty high^47^,^48^. Self-report of physician diagnosis of stroke might be valid instrument for large epidemiological survey. Consider that the relatively low education level and thus poor public knowledge of stroke for the participants in this study, the self-reported stroke might include the reporting of other stroke-like or stroke-related conditions. On the other hand, participants with mild stroke might tend to under-report the disease. Moreover, there is no specific classification of stroke, because stroke outcome in the present study was assessed through self-report. Therefore, we could not evaluate the associations between parity and the risks of different stroke subtypes. Third, some seriously ill patients with stroke could not participate in the medical examination, and consequently their corresponding information may be missed. Fourth, we did not collect data on some other potential confounders, such as income. Socioeconomic status is known to be related to both number of children and stroke risk, and its confounding effect should be a major concern in evaluating the association between parity and stroke. In our study, we found that adjusting for education attenuated, but did not eliminate, the association. Education has an apparent protective effect on the relationship between parity and stroke risk. We did not collect any other information on socioeconomic status, such as income, and thus we could not ideally evaluate its effects on the results. Additionally, we did not collect information on pregnancy complications and were therefore unable to account for their potential effects on the results. Pregnancy complications such as gestational diabetes, pre-eclampsia, and eclampsia have been associated with an elevated risk of stroke in later life^34^,^49^. Finally, the findings of our study, conducted among middle-aged and older Chinese women, may not be generalizable to other populations.

In conclusion, parity is associated with the risk of stroke independent of possible confounders. Our findings have significant implications for further longitudinal studies, in which pregnancy complications, more socioeconomic information, and changes in physiology and lifestyle during pregnancy and the postpartum period can be prospectively measured to confirm the effects between parity and stroke. Further studies are also very much needed to explore the unclear mechanism underlying the link between parity and stroke.

## Methods

### Study participants

Data for the analysis was derived from the base-line survey of Dongfeng-Tongji Cohort Study, which is a collaborative study between Tongji Medical College, Huazhong University of Science and Technology, and Dongfeng Motor Corporation. The rational, design, fundamentals, and methods of the Dongfeng-Tongji Cohort Study have been described in detail previously^50^. Briefly, 87% (27,009 out of 31,000) of retired employees at Dongfeng Motor Corporation were recruited and agreed to participate in the study during September 2008 to June 2010. A structured questionnaire was used to collect information on demographic characteristics, lifestyle, disease history, reproduction-related information by trained interviewers. All participants were required to have a medical examination and provide biological samples such as blood, urine.

Among the 27,009 eligible participants, 14,957 were women. For current analysis, we excluded women who never had a live birth (n = 205). The participants who had missing data on parity or on self-reporting stroke were also excluded. All together, 680 participants were excluded from our study. The final sample size for the study was 14,277 women.

### Ethics Statement

Written informed consent was obtained from all the participants. The study was approved by the Medical Ethics Committee of the School of Public Health, Tongji Medical College, Huazhong University of Science and Technology and Dongfeng General Hospital. All the methods in the present study were carried out in accordance with the approved guidelines.

### Assessment of Stroke

Individuals with stroke in this study were the survivors from nonfatal stroke. The occurrence of stroke was ascertained by asking the following question: “have you suffered a stroke that was confirmed by a physician?” Every positive response was written down clearly. The patients who suffer aphasia or vascular dementia after stroke may be disabled to describe their medical history. In this situation, their family member was asked to help them to answer the questionnaire in our current study. In the current study, we did not distinguish between ischemic stroke and hemorrhagic stroke.

### Assessment of Parity

Parity was defined as the self-reported total number of live births. In this study, parity was classified into four categories: one live birth, two live births, three live births, and four or more live births.

### Assessment of covariates

The questionnaire collected demographic information on sex, age, education and marital status (married, unmarried, widowed, divorced). Lifestyle information on smoking status (smoke more than 1 cigarette per day in the previous 6 months), passive smoking (exposure to environmental tobacco smoke for more than 15 minutes per day and more than one day per week over the past 6 months), alcohol drinking status (drink more than once per week in the previous 6 months), and physical activity was also obtained. Physical activity was defined as those who practiced walking, biking, tai chi, jogging, swimming, dancing, climbing stairs, playing ball sports more than 20 minutes per day and more than three times per week over the past 6 months. A positive family history of stroke was defined as the self-reported of at least one first-degree family member (father, mother, siblings, or offspring) being diagnosed by physician as having stroke. The reproduction related information including history of delivery, abortions, menopausal status (pre-menopause, post-menopause), age at menopause, history of contraceptive use, and history of hormone replacement therapy (HRT) were also collected. The anthropometric data included weight, height, waist circumference and systolic and diastolic blood pressures. Results of laboratory tests were obtained by medical examination. The BMI (body mass index) was calculated as the ratio of the weight in kilograms divided by height squared in meters.

### Statistical analysis

The analysis of variance (ANOVA) was used to test differences in means among four categories of parity for the numerical variables, and chi-square tests were used for categorical variables. Categorical data were summarized as proportion (%) and numerical variables were described as mean ± SD. We regarded stroke as a dependent variable and a series of multivariable logistic regression models were used to calculate the odds ratios (ORs) and 95% confidence intervals (95% CIs) across parity categories. Women with one live birth were regarded as the reference category. Model 1 examined the relationship between parity and stroke without adjustment for any covariates. Model 2 included education, marital status, smoking status, passive smoking status, alcohol drinking status, physical activity, family history of stroke, diabetes, hypertension, and factors related to reproductive health including menopausal status, age at menopause, use of contraceptives, and hormone replacement therapy (HRT). Model 3 included the covariates in Model2 plus abortion. Model 4 included the covariates in Model 3 plus age. At last, Model 5, the fully adjusted model, included all the above mentioned potential confounders in Model 4 plus BMI. The Cochran-Armitage trend tests were conducted using SAS statistical software (version 9.4) and other statistical analyses were performed by using SPSS software (version 17.0). All reported *P* values were two-sided, and *P* < 0.05 was considered to be statistically significant. The association between parity and prevalent stroke was assessed graphically according to age-adjusted model and fully adjusted model, separately, in the current study.

## Additional Information

**How to cite this article**: Zhang, Y. *et al.* Parity and Risk of Stroke among Chinese Women: Cross-sectional Evidence from the Dongfeng-Tongji Cohort Study. *Sci. Rep.* **5**, 16992; doi: 10.1038/srep16992 (2015).

## Figures and Tables

**Table 1:** Baseline characteristics of study participants of 14,277 women by parity.

**Parity**						
Characteristics	1(n = 4949)	2(n = 4781)	3(n = 2793)	≥4(n = 1754)	χ^2^/F	*P*
Age (y) (means ± SD)	54.19 ± 5.11	60.35 ± 5.78	65.32 ± 6.20	70.30 ± 6.15	4397.71	<0.001**
Marital status					795.23	<0.001*
Unmarried (%)	7(0.1)	11(0.2)	5(0.2)	1(0.1)		
Married (%)	4493(91.0)	4172(87.6)	2324(83.3)	1268(72.4)		
Widowed (%)	236(4.8)	459(9.6)	432(15.5)	476(27.2)		
Divorced (%)	201(4.1)	120(2.5)	29(1.0)	7(0.4)		
Education					2855.35	<0.001*
Elementary or below (%)	496(10.1)	1312(27.6)	1272(46.0)	1169(68.1)		
Junior high school (%)	1887(38.4)	1932(40.7)	971(35.1)	430(25.0)		
High school (%)	2024(41.2)	1150(24.2)	425(15.4)	96(5.6)		
College or above (%)	510(10.4)	355(7.5)	97(3.5)	22(1.3)		
Smoking status					340.87	<0.001*
Never (%)	4839(98.8)	4641(97.8)	2658(95.9)	1578(90.4)		
Current (%)	48(1.0)	84(1.8)	75(2.7)	110(6.3)		
Former (%)	12(0.2)	18(0.4)	38(1.4)	58(3.3)		
Alcohol consumption					13.52	0.035*
Never (%)	4552(92.0)	4438(92.9)	2603(93.2)	1637(93.3)		
Current (%)	348(7.0)	286(6.0)	151(5.4)	97(5.5)		
Former (%)	46(0.9)	54(1.1)	38(1.4)	20(1.1)		
Passive smoking (%)	1223(24.7)	958(20.1)	482(17.3)	245(14.0)	118.33	<0.001*
Family history of stroke (%)	397(8.1)	292(6.2)	79(2.8)	38(2.2)	138.00	<0.001*
Menopause status (%)	3927(79.4)	4558(95.4)	2725(97.6)	1722(98.2)	1143.58	<0.001*
History of contraceptive use (%)	1238(25.0)	1210(25.4)	614(22.0)	274(15.7)	79.25	<0.001*
History of HRT (%)	193(3.9)	160(3.4)	59(2.1)	24(1.4)	38.61	<0.001*
Hypertension (%)	1746(36.3)	2332(50.0)	1689(61.5)	1140(66.6)	695.97	<0.001*
Diabetes (%)	313(6.3)	562(11.8)	489(17.5)	349(19.9)	335.33	<0.001*
Physical activity (%)	4253(88.1)	4179(87.9)	2466(88.5)	1544(88.2)	0.52	0.92*
Stroke (%)	59(1.2)	106(2.2)	123(4.4)	92(5.2)	122.81	<0.001*
Menopause age	48.71 ± 3.87	49.24 ± 3.69	49.13 ± 3.73	49.10 ± 3.84	14.68	<0.001**
Abortion	1.33 ± 1.20	1.08 ± 1.12	0.92 ± 1.07	0.76 ± 1.03	143.87	<0.001**
BMI (kg/m^2^)	23.85 ± 3.31	24.55 ± 3.42	25.20 ± 3.62	25.20 ± 3.92	115.18	<0.001**

Note: Abbreviations: y, years; BMI, body mass index; HRT, hormone replacement therapy.

Variables are described as mean ± SD for numerical data and proportion (%) for categorical data.

^*^Chi-square test.

^**^ANOVA test.

**Table 2:** Odds ratios (95% confidence intervals) of stroke according to parity categories in different logistic regression models.

parity	Model 1	Model 2	Model 3	Model 4	Model 5
1	1.00	1.00	1.00	1.00	1.00
2	1.88(1.36–2.59)	1.36(0.95–1.95)	1.36(0.94–1.95)	1.24(0.86–1.81)	1.24(0.85–1.81)
3	3.82(2.79–5.23)	2.28(1.58–3.31)	2.31(1.59–3.34)	1.98(1.31–2.98)	1.97(1.30–2.98)
≥4	4.59(3.29–6.39)	2.28(1.52–3.44)	2.31(1.53–3.48)	1.87(1.16–3.04)	1.86(1.14–3.03)

Note: Model 1: univariate.

Model 2 adjusted for education, marital status, smoking status, alcohol consumption, passive smoking, family history of stroke, menopause status, history of contraceptive use, history of hormone replacement therapy, hypertension, diabetes, physical activity, menopause age.

Model 3 added abortion for adjusting based on Model 2.

Model 4 added age for adjusting based on Model 3.

Model 5 added BMI for adjusting based on Model 4.

Abbreviation: CI, confidence interval; BMI, body mass index.

## References

[b1] Feigin V. L. *et al.* Global and regional burden of stroke during 1990–2010: findings from the Global Burden of Disease Study 2010. Lancet 383, 245–254 (2014). 24449944 10.1016/s0140-6736(13)61953-4 PMC4181600

[b2] *Health statistics and health information systems.* (2012) Available at: http://www.who.int/healthinfo/global_burden_disease/estimates/en/. (Date of access:19/08/ 2015).

[b3] GBD 2010 Arrow Diagram. (2010) Available at: http://vizhub.healthdata.org/irank/arrow.php. (Date of access: 19/08/2015).

[b4] Reeves M. J. *et al.* Sex differences in stroke: epidemiology, clinical presentation, medical care, and outcomes. Lancet Neurol. 7, 915–926 (2008). 18722812 10.1016/S1474-4422(08)70193-5 PMC2665267

[b5] Gunderson E. *et al.* Excess gains in weight and waist circumference associated with childbearing: The Coronary Artery Risk Development in Young Adults Study (CARDIA). Int. J. Obes. Relat. Metab. Disord. 28, 525–535 (2004). 14770188 10.1038/sj.ijo.0802551 PMC3133634

[b6] Lawlor D. A. *et al.* Is the association between parity and coronary heart disease due to biological effects of pregnancy or adverse lifestyle risk factors associated with child-rearing? Findings from the British Women’s Heart and Health Study and the British Regional Heart Study. Circulation 107, 1260–1264 (2003). 12628945 10.1161/01.cir.0000053441.43495.1a

[b7] Ness R. B. *et al.* Number of pregnancies and the subsequent risk of cardiovascular disease. N. Engl. J. Med. 328, 1528–1533 (1993). 8267704 10.1056/NEJM199305273282104

[b8] Qureshi A. I., Giles W. H., Croft J. B. & Stern B. J. Number of pregnancies and risk for stroke and stroke subtypes. Arch. Neurol. 54, 203–206 (1997). 9041862 10.1001/archneur.1997.00550140073015

[b9] Zhang X. *et al.* Pregnancy, childrearing, and risk of stroke in Chinese women. Stroke 40, 2680–2684 (2009). 19461027 10.1161/STROKEAHA.109.547554 PMC2737806

[b10] Gaist D., Pedersen L., Cnattingius S. & Sørensen H. T. Parity and Risk of Subarachnoid Hemorrhage in Women A Nested Case-Control Study Based on National Swedish Registries. Stroke 35, 28–32 (2004). 14657450 10.1161/01.STR.0000105933.16654.B4

[b11] Yang C.-Y., Chang C.-C., Kuo H.-W. & Chiu H.-F. Parity and risk of death from subarachnoid hemorrhage in women: evidence from a cohort in Taiwan. Neurology 67, 514–515 (2006). 16894119 10.1212/01.wnl.0000227938.06750.ec

[b12] Bazzano L. A. *et al.* Body mass index and risk of stroke among Chinese men and women. Ann Neurol. 67, 11–20 (2010). 20186847 10.1002/ana.21950 PMC4371851

[b13] Bonita R. *et al.* The global stroke initiative. Lancet Neurol. 3, 391–393 (2004). 15207791 10.1016/S1474-4422(04)00800-2

[b14] Boysen G. *et al.* Stroke incidence and risk factors for stroke in Copenhagen, Denmark. Stroke 19, 1345–1353 (1988). 3188119 10.1161/01.str.19.11.1345

[b15] Hu F. B. *et al.* Physical activity and risk of stroke in women. JAMA 283, 2961–2967 (2000). 10865274 10.1001/jama.283.22.2961

[b16] Jung S.-Y., Bae H.-J., Park B.-J. & Yoon B.-W. Parity and risk of hemorrhagic strokes. Neurology 74, 1424–1429 (2010). 20335561 10.1212/WNL.0b013e3181dc13a5

[b17] Yang L. *et al.* Reproductive history, oral contraceptive use, and the risk of ischemic and hemorrhagic stoke in a cohort study of middle-aged Swedish women. Stroke 40, 1050–1058 (2009). 19211494 10.1161/STROKEAHA.108.531913

[b18] Nakayama H., Jørgensen H., Raaschou H. & Olsen T. The influence of age on stroke outcome. The Copenhagen Stroke Study. Stroke 25, 808–813 (1994). 8160225 10.1161/01.str.25.4.808

[b19] Park J. W., Lee S. Y., Kim S. Y., Choe H. & Jee S. H. BMI and stroke risk in Korean women. Obesity 16, 396–401 (2008). 18239650 10.1038/oby.2007.67

[b20] Liu J., Larsen U. & Wyshak G. Prevalence of primary infertility in China: in-depth analysis of infertility differentials in three minority province/autonomous regions. J. Biosoc. Sci. 37, 55–74 (2005). 15688571 10.1017/s0021932003006461

[b21] Bernstein L. *et al.* Estrogen and sex hormone-binding globulin levels in nulliparous and parous women. J. Natl. Cancer Inst. 74, 741–745 (1985). 3857369

[b22] Cole P., MacMahon B. & Brown J. B. Oestrogen profiles of parous and nulliparous women. Lancet 2, 596–599 (1976). 61341 10.1016/s0140-6736(76)90666-8

[b23] Liu J., Larsen U. & Wyshak G. Prevalence of primary infertility in China: in-depth analysis of infertility differentials in three minority province/autonomous regions. J. Biosoc. Sci. 37, 55–74 (2005). 15688571 10.1017/s0021932003006461

[b24] Beral V. Long term effects of childbearing on health. J. Epidemiol. Community Health 39, 343–346 (1985). 4086966 10.1136/jech.39.4.343 PMC1052469

[b25] Humphries K. H. *et al.* Parity and Carotid Artery Atherosclerosis in Elderly Women The Rotterdam Study. Stroke 32, 2259–2264 (2001). 11588310 10.1161/hs1001.097224

[b26] Wolff B. *et al.* Relation of parity with common carotid intima-media thickness among women of the study of health in Pomerania. Stroke 36, 938–943 (2005). 15845891 10.1161/01.STR.0000162712.27799.20

[b27] Okamoto K. *et al.* Menstrual and Reproductive Factors for Subarachnoid Hemorrhage Risk in Women A Case-Control Study in Nagoya, Japan. Stroke 32, 2841–2844 (2001). 11739984 10.1161/hs1201.099383

[b28] Mhurchu C. N., Anderson C., Jamrozik K., Hankey G. & Dunbabin D. Hormonal Factors and Risk of Aneurysmal Subarachnoid Hemorrhage An International Population-Based, Case-Control Study. Stroke 32, 606–612 (2001). 11239175 10.1161/01.str.32.3.606

[b29] Davie C. & O’Brien P. Stroke and pregnancy. J. Neurol. Neurosurg. Psychiatry 79, 240–245 (2008). 17986502 10.1136/jnnp.2007.116939

[b30] Feske S. K. Stroke in pregnancy. Semin. Neurol. 27, 442–452 (2007). 17940923 10.1055/s-2007-991126

[b31] Kittner S. J. *et al.* Pregnancy and the risk of stroke. N. Engl. J. Med. 335, 768–774 (1996). 8703181 10.1056/NEJM199609123351102 PMC1479545

[b32] Juntunen K., Kirkinen P. & Kauppila A. The clinical outcome in pregnancies of grand grand multiparous women. Acta Obstet. Gynecol. Scand. 76, 755–759 (1997). 9348253 10.3109/00016349709024342

[b33] Bellamy L., Casas J. P., Hingorani A. D. & Williams D. J. Pre-eclampsia and risk of cardiovascular disease and cancer in later life: systematic review and meta-analysis. BMJ 335, 974 (2007). 17975258 10.1136/bmj.39335.385301.BE PMC2072042

[b34] Wilson B. J. *et al.* Hypertensive diseases of pregnancy and risk of hypertension and stroke in later life: results from cohort study. BMJ 326, 845 (2003). 12702615 10.1136/bmj.326.7394.845 PMC153466

[b35] Grundy E. & Tomassini C. Fertility history and health in later life: a record linkage study in England and Wales. Soc. Sci. Med. 61, 217–228 (2005). 15847974 10.1016/j.socscimed.2004.11.046

[b36] Castelao J. E. & Gago-Dominguez M. Risk factors for cardiovascular disease in women: relationship to lipid peroxidation and oxidative stress. Med. Hypotheses 71, 39–44 (2008). 18308480 10.1016/j.mehy.2007.10.016

[b37] Skilton M. R., Sérusclat A., Begg L. M., Moulin P. & Bonnet F. Parity and carotid atherosclerosis in men and women: insights into the roles of childbearing and child-rearing. Stroke 40, 1152–1157 (2009). 19211493 10.1161/STROKEAHA.108.535807

[b38] Kvåle G., Heuch I. & NILSSEN S. Parity in relation to mortality and cancer incidence: a prospective study of Norwegian women. Int. J. Epidemiol. 23, 691–699 (1994). 8002181 10.1093/ije/23.4.691

[b39] Linné Y., Barkeling B. & Rössner S. Long‐term weight development after pregnancy. Obes Rev. 3, 75–83 (2002). 12120423 10.1046/j.1467-789x.2002.00061.x

[b40] Do Lee C., Folsom A. R. & Blair S. N. Physical activity and stroke risk a meta-analysis. Stroke 34, 2475–2481 (2003). 14500932 10.1161/01.STR.0000091843.02517.9D

[b41] Wang W. *et al.* [Gestational weight gain and its relationship with the birthweight of offspring]. Zhonghua fu chan ke za zhi 48, 321–325 (2013). 24016471

[b42] Wong W., Tang N. L., Lau T. & Wong T. A new recommendation for maternal weight gain in Chinese women. J. Am. Diet. Assoc. 100, 791–796 (2000). 10916517 10.1016/S0002-8223(00)00230-3

[b43] Liu M. *et al.* Stroke in China: epidemiology, prevention, and management strategies.Lancet Neurol. 6, 456–464 (2007). 17434100 10.1016/S1474-4422(07)70004-2

[b44] Grinblatt J. A. Number of pregnancies and risk of cardiovascular disease. N. Engl. J. Med. 329, 1894–1895 (1993). 8247047 10.1056/NEJM199312163292515

[b45] Zhao D. *et al.* Epidemiological transition of stroke in China: twenty-one–year observational study from the Sino-MONICA-Beijing Project. Stroke 39, 1668–1674 (2008). 18309149 10.1161/STROKEAHA.107.502807

[b46] Fu C., Zhang L. & Li Y. Characteristics of the changes of population fertility in China based on the Sixth Census. Statistical Research 30, 68–75 (2013).

[b47] Okura Y., Urban L. H., Mahoney D. W., Jacobsen S. J. & Rodeheffer R. J. Agreement between self-report questionnaires and medical record data was substantial for diabetes, hypertension, myocardial infarction and stroke but not for heart failure. J. Clin. Epidemiol. 57, 1096–1103 (2004). 15528061 10.1016/j.jclinepi.2004.04.005

[b48] Machón M. *et al.* Validity of self-reported prevalent cases of stroke and acute myocardial infarction in the Spanish cohort of the EPIC study. J. Epidemiol. Community Health 67, 71–75 (2013). 22577182 10.1136/jech-2011-200104

[b49] Carpenter M. W. Gestational diabetes, pregnancy hypertension, and late vascular disease. Diabetes Care 30 Suppl 2, S246–250, doi: 10.2337/dc07-s224 (2007). 17596480

[b50] Wang F. *et al.* Cohort Profile: the Dongfeng-Tongji cohort study of retired workers. Int. J. Epidemiol. 42, 731–740 (2013). 22531126 10.1093/ije/dys053

